# Generation and characterization of human monoclonal single chain variable fragments (scFvs) against envelope third variable region (V3) of HIV-1 clade C

**DOI:** 10.1186/1471-2334-12-S1-P11

**Published:** 2012-05-04

**Authors:** Rajesh Kumar, Raiees Andrabi, Ashutosh Tiwari, Somi Sankaran Prakash, Naveet Wig, Durgashree Dutta, Anurag Sankhyan, Lubina Khan, Subrata Sinha, Kalpana Luthra

**Affiliations:** 1 All India Institute of Medical Sciences, New Delhi, India; 2 Director, National Brain Research Centre, Manesar, India

## Background

Production of human monoclonal antibodies with broad neutralizing activity is an essential part of HIV-1 prophylactic vaccine. Majority of the viruses infecting Indian patients belong to clade C.

## Methods

A phage library of 7000 clones was constructed from a drug naive HIV-1 clade C infected Indian patient whose plasma exhibited high potential neutralizing potential against a panel of viruses and also displayed cross-reactive anti-V3 antibodies. PBMCs were isolated and EBV transformed. Cells (wells) producing anti-V3 antibodies were preselected with V3-CTB fusion protein and expanded. Total RNA was isolated and cDNA was constructed followed by VH and VL amplification. scFvs were constructed, cloned into phagemid vector and expressed in Escherichia coli. We assessed the expression of the scFvs by SDS-PAGE and Western blotting. Specificity was examined by ELISA.

## Results

A total of 30 clones were randomly selected after biopanning and checked for their binding to V3 peptides of clade C and B. Ten clones showed binding in phage ELISA, 8 were cross-reactive to both the V3 peptides while the other 2 were specific to V3C. The clones did not show cross-reactivity against other unrelated peptides. The recombinant anti-V3 scFvs (32kD) were expressed and confirmed by SDS-PAGE and Western blotting. DNA fingerprinting analysis showed that 9 out of the 10 clones were distinct.

## Conclusion

This is the first report on the generation of human anti-V3 scFvs against HIV-1 clade C. Further assessment of the neutralization efficiency of these scFvs would reveal their potential for passive immunotherapy.

